# Comparison of Physicochemical Properties of Fly Ash Precursor, Na-P1(C) Zeolite–Carbon Composite and Na-P1 Zeolite—Adsorption Affinity to Divalent Pb and Zn Cations

**DOI:** 10.3390/ma14113018

**Published:** 2021-06-02

**Authors:** Rafał Panek, Magdalena Medykowska, Katarzyna Szewczuk-Karpisz, Małgorzata Wiśniewska

**Affiliations:** 1 Department of Geotechnical Engineering, Faculty of Civil Engineering and Architecture, Lublin University of Technology, Nadbystrzycka 40, 20-618 Lublin, Poland; 2 Department of Radiochemistry and Environmental Chemistry, Faculty of Chemistry, Maria Curie-Sklodowska University in Lublin, M. Curie-Sklodowska Sq. 3, 20-031 Lublin, Poland; wisniewska@hektor.umcs.lublin.pl; 3 Institute of Agrophysics, Polish Academy of Sciences, Doświadczalna 4, 20-290 Lublin, Poland; k.szewczuk-karpisz@ipan.lublin.pl

**Keywords:** Na-P1 zeolite, zeolite–carbon composite, zinc and lead adsorption, simultaneous ions removal, desorption by HCl/NaOH, electrokinetic parameters

## Abstract

Considering the growing needs of environmental remediation, new effective solutions should be sought. Therefore, the adsorbed amounts of heavy metal ions, such as lead(II) and zinc(II), on the surface of high-carbon fly ash (HiC FA), zeolite-–carbon composite (Na-P1(C)) and pure zeolite (Na-P1), were investigated. The applied solids were characterized using the following techniques: XRD, SEM-EDS, TEM, porosimetry, SLS, electrophoresis and potentiometric titration. The heavy metal concentration in the probes was determined by applying ICP-OES spectroscopy. Adsorption/desorption and electrokinetic measurements were performed in the systems containing one or two adsorbates. The obtained results indicated that Pb(II) ions are adsorbed in larger amounts on the investigated solid surface due to the molecular sieving effect. The largest adsorption capacity relative to lead(II) ions was observed for pure Na-P1 zeolite (407 mg/g). The simultaneous presence of Pb(II) + Zn(II) mixed adsorbates minimally affects the amount of adsorbed Pb(II) ions and causes a significant decrease of Zn(II) ion adsorption (in comparison with analogous systems containing single adsorbates). It was also shown that all solids can be efficiently regenerated using hydrochloric acid. Thus, the selected pure zeolite can be successfully applied in soil remediation or other purifying technologies as an effective Pb(II) adsorbent.

## 1. Introduction

Human activities and their interactions with the environment, such as mining, oil and gas quarrying, have a significant impact on the progressive destruction and pollution of natural ecosystems [1]. Increased soil exploitation related to attempts to provide food for the still significantly increasing world population led to soil degradation [2,3]. Therefore, it is important to look for as many solutions as possible to minimize the results of adverse processes occurring in soil. Organic amendments, such as livestock manure, biosolids, pulp and paper mill by-products, etc. [4], have been used successfully. Biochar amendments, green waste compost and triple superphosphate (TSP) as well as clay minerals, such as kaolinite [5], were applied to reduce the bioavailability of heavy metals in soils [6,7]. Another way to improve soil quality is zeolites, which have been successfully used as an additive for the composting of organic solid waste. This allowed for a shorter composting period, a reduction in greenhouse gas and ammonia emissions, as well as a reduction in the total amount and bioavailability of heavy metals. The use of zeolite-modified compost contributed to better yields, water retention and reduced loss of nutrients [8].

Zeolites are minerals, formed during hydrothermal processes (natural zeolites) or chemical processes (synthetic zeolites). They are crystalline, porous hydrated aluminosilicates of sodium and calcium, less frequently of strontium or barium. Their special properties include not only the ion-exchange ability and significant resistance to acid and high temperature, but also the possibility of removing water from the tubules (zeolite water) without disturbing the structure of the zeolite. Other unique properties are low density, catalytic properties and a large volume of free space. Zeolites also are capable of ion and molecule sorption [9,10]. Some zeolites are present in the soils, such as the most common clinoptilolite, but also chabazite, laumonite, analcime and stilbite [11,12]. Currently, synthetic zeolites are more and more widely used materials. They are applied due to physical and chemical properties and are more uniform and predictable characteristics than natural zeolites [13]. 

Zeolites are widely used as soil fertilizers, being a good alternative to chemicals. Zeolite-based fertilizers have the ability to reduce the nutrient release, which contributes to better nourishment of the soil and does not pose a threat of increased nitrogen, phosphorus or potassium concentrations in surface water. Surfactant-modified zeolite was used as a fertilizer carrier that makes phosphorus release slower [14]. The addition of nanoporous zeolite also increased the nitrogen bioavailability from urea fertilizers [15]. Zeolite fertilizers can be synthesized from coal ash, which is a problematic industrial by-product [16]. In remediation of heavy-metal-contaminated soils, both natural and synthetic zeolites may be applied [17,18].

The presence of heavy metals in the environment, i.e., in waters or soils, due to their properties (toxicity, stability, possibility of accumulation or carcinogenicity) is one of the most important issues that we have to deal with. On the other hand, the presence of certain metals in a given environment may be a benefit, e.g., zinc as a representative of microelements is necessary in sufficient quantities for the growth of plants, but its excess in soil inhibits their growth, disrupting their development and metabolism [19]. Lead is absolutely toxic to plants and is not needed for their growth. It threatens the life and health of animals and humans [20]. Therefore, it is important to develop new materials enabling the removal of heavy metals from the environment. Based on the performed study, it can be stated whether selected adsorbents can be successfully used in water purification and soil remediation. Hui et al. proved that both zeolite (Na-A) and zeolite-–carbon composite made from coal gangue can be useful for Cu^2+^ and rhodamine B removal from a water solution [21]. In turn, Wanyonyi et al. described the influence of zeolite pore topology and chemistry, claiming that the high diameter of pores exhibited appreciable loading of the cations [22]. Kragović et al. [23] tested natural zeolite (NZA) and zeolite modified via alginate (FRA), whereas Abdelrahman et al. [24] used rice husks and aluminum can waste to produce geopolymer–zeolite products. The studies in which the natural zeolite clinoptilolite showed a similar ability to remove heavy metals (As^5+^, Cu^2+^, Ni^2+^, Pb^2+^ and Zn^2+^) as commercially available γ-Al_2_O_3_ have also been reported [25]. Due to the fact that inorganic and organic substances significantly affect the structure of the electrical double layer, the influence of adsorbates on the zeta potential and surface charge of zeolites and zeolite–carbon composites should also be determined [26,27].

This paper describes the synthesis and characterization of a novel Na-P1 zeolite and its Na-P1(C) zeolite–carbon composite as adsorbents reducing the bioavailability of heavy metals (zinc and lead) to plants and animals in soil. These adsorbents were tested in single and mixed systems of heavy metal ions to understand the influences of particular adsorbates on the adsorption process. In addition, high-carbon fly ash (HiC FA) was also characterized as a substrate for the hydrothermal reaction, leading to the formation of the above adsorbents [28]. To our knowledge, for the first time, a waste-based sorbent in the form of high-carbon fly ash was used for heavy metal removal, which, due to its chemical composition, cannot be used in civil engineering (the main branch of fly ash disposal). Additionally, undoubtedly, novelty is a scale of research conducted. A semi-technical technology line was used to synthesize both sorbents, which can ease application in industry in the near future.

## 2. Materials and Methods

### 2.1. Materials

The preparation of the materials was started by obtaining the Na-P1(C) zeolite–carbon composite. Fly ash, the starting material from the Janikowo Thermal Power Plant resulting from the conventional combustion of hard coal, was used in this procedure. Due to its electromagnetic separation, the obtained high-carbon product (HiC FA) could be used to produce a carbon–zeolite composite. The final product was created in a typical zeolitization process based on the hydrothermal reaction of fly ash with an aqueous solution of sodium hydroxide [29,30].

The Na-P1(C) material was prepared on a technological line for the synthesis of ash zeolites. At the first stage, 20 kg of high-carbon ash was mixed with 90 dm^3^ of 3 M NaOH solution. Then the whole mixture was stirred with a mechanical stirrer at a temperature of 90 °C for 24 h. At the final stage the obtained product was washed, rinsed and dried at 105 °C. This reaction also resulted in the production of a waste solution rich in silicon and aluminum coming from the dissolution of the ash aluminosilicate glaze and sodium coming from sodium hydroxide [31,32]. This waste was a substrate for the synthesis of the second type of material, which was pure Na-P1 zeolite without ash residue. It was prepared on a technological scale using the following steps: 40 dm^3^ of waste solution was mixed with 10 dm^3^ of 2 M aqueous NaOH solution, where 80 g of aluminum foil was dissolved. The mixture was left for 48 h at 100 °C. In the next stage, the obtained product was filtered, washed and dried at a temperature of 105 °C. The materials prepared in this way were characterized in terms of mineralogy, structure and texture.

### 2.2. Methods

The solids characterization was performed as follows. The phase composition of the solids was characterized by X-ray diffraction in the Panalytical XPert Pro MPD(Eindhoven, The Netherlands) apparatus with a copper lamp (CuK_α_ = 1.54178 Å). The angular range was 5–65° 2Θ with a step of 0.02° 2θ lasting 5 s. The X’Pert Highscore software ver. 4.1 (Almelo, The Netherlands) was used to process the diffraction data. The mineral phases were identified using the PDF-2 release 2010 database formalized by JCPDS-ICDD. 

The Panalytical Epsilon 3 spectrometer, equipped with an X-ray tube Rh 9 W, 50 kV, 1 mA, 4096 channel spectrum analyzer and a high-resolution semiconductor SDD detector cooled by a Peltier cell, was used to determine the elemental composition. The obtained results considered the LOI (loss of ignition). 

The Malvern Mastersizer 3000 apparatus allowed the estimation of the grain size through the phenomenon of laser diffraction. The experiment was carried out in the aquatic environment in the HYDRO EV attachment. 

The FEI Quanta 250 FEG scanning electron microscope equipped with an EDS attachment from the EDAX company (Mahwah, NJ, USA) allowed the morphological analysis. The experiment was carried out on the samples sprayed with a conductive carbon layer at an accelerating voltage of 15 keV. 

The HR/TEM analysis of the Na-P1 sample was conducted on an electron microscope, Titan G2 60–300 kV (FEI Company, Hillsboro, OR, USA), applying an accelerating voltage of the electron beam equal to 300 kV.

The ASAP apparatus supplied by Micromeritics Instrument Corporation was used to measure textural parameters (the test was carried out using a low-temperature nitrogen adsorption/desorption isotherm in liquid nitrogen at a temperature of 77 K (−194.85 °C) in the range of relative pressures p/p_0_ ranging from 1.5 × 10^−7^ to 0.99). In turn, the analysis of the shape of nitrogen vapor adsorption isotherms was characterized by IUPAC classification [33,34]. Samples for N_2_ adsorption were prepared by degassing at 300 °C for 12 h under reduced pressure in the degassing port. Then the samples were degassed at the analytical port just before analysis for 4 h at 300 °C.

The adsorbed amount determination of heavy metal ions on the surface of HiC FA, Na-P1 and Na-P1(C) was based on a static method and the following equation (Equation (1)) [35]:

(1) $$\Gamma =\frac{c_{\mathrm{ads}}\cdot V}{m}$$

where *c_ads_*—the heavy metal adsorbed concentration (the difference in the heavy metal concentration in the system before and after its adsorption), *V*—the suspension volume, and *m*—the solid weight. 

The samples were prepared using 0.003 g of HiC FA, Na-P1 or Na-P1(C), which was added to the solutions containing the supporting electrolyte (0.001 M NaCl) and the selected heavy metal ions with the concentrations of 10, 50, 70, 100, 150 and 200 ppm (total volume of solution was 10 cm^3^). In the case of mixed adsorbate systems, the concentration of both ions was 100 ppm. The pH of all samples was set to the value of 5 using 0.1 M HCl, 0.1 M NaOH and a pH meter supplied by Beckman (Brea, CA, USA). After the adsorption process, which lasted 3 h, the solid was separated from the solution by paper filters. The time of the adsorption process was established based on the kinetics study. The concentration of ions in supernatants was determined using optical emission spectrometry with inductively coupled plasma (Thermo Scientific iCAP™ 7200 ICP-OES analyzer). Based on the obtained results, the adsorption isotherms of Zn(II) and Pb(II) on the surface of HiC FA, Na-P1 and Na-P1(C) were prepared. The experimental data were fitted to the selected theoretical models, i.e., Langmuir (Equation (2)) and Freundlich (Equation (3)) [36,37]:

(2) $$q_{e}=\frac{q_{m}K_{L}C_{e}}{1+K_{L}C_{e}}$$

(3) $$q_{e}=K_{F}C_{e}^{1/n}$$

where *K_F_* and *K_L_*—the Freundlich [mg/g (mg/L)^−1/nF^] and Langmuir [dm^3^/mg] parameters, respectively, *q_e_*—the equilibrium adsorption capacity [mg/g], *C_e_*—the equilibrium liquid phase concentration [mg/dm^3^], *q_m_*—the maximum adsorption capacity in a Langmuir model [mg/g], and *n*—the Freundlich constant related to adsorption intensity.

The samples for **adsorption kinetics study** were prepared by adding 0.003 g of the selected adsorbent to 10 cm^3^ of solutions containing the supporting electrolyte (0.001 M NaCl) and heavy metal ions with a concentration of 100 ppm. Then the pH was adjusted to the value of 5 and adsorption was performed for 10, 30, 60, 90, 120 and 180 min. After that step, the solids were separated by paper filters and the concentration of heavy metal ions in supernatants was determined by the ICP-OES technique. The modeling of the results was made using the equations of pseudo-first order (Equation (4)) and pseudo-second order (Equation (5)) [38,39,40,41]:

(4) $$\frac{dq_{t}}{dt}=k_{2}\left(q_{e}-q_{t}\right)$$

(5) $$\frac{dq_{t}}{dt}=k_{2}{\left(q_{e}-q_{t}\right)}^{2}$$

where *q_e_*—the adsorbed amount at equilibrium [mg/g], *q_t_*—the adsorbed amount after time ‘*t*’ [mg/g], and *k*_1_ [1/min] and *k*_2_ [g/mg·min]—the equilibrium rate constants.

**Desorption measurements** began with the preparation of samples containing a supporting electrolyte (0.001 M NaCl), 0.003 g of HiC FA, Na-P1 or Na-P1(C) and heavy metal ions at concentrations of 100 ppm (total volume of solution was 10 cm^3^). Then, the pH value was adjusted to 5 and the adsorption was carried out for 3 h. The solid was separated from the solution by paper filters and then 10 cm^3^ of 0.1 M HCl or NaCl was added to the separated solids. The desorption process lasted 1 h, after which the solid was separated again using paper filters and the concentration of ions in supernatants was determined by means of ICP-OES. Desorption degree (*D*, %) was calculated based on the equation (Equation (6)):

(6) $$D=\frac{Cd\;}{\;Ca}\cdot 100\%$$

where *C_d_*—the amount of desorbed heavy metal ions, and *C_a_*—the amount of absorbed heavy metal ions.

**Potentiometric titration** was used to determine the surface charge density (σ_0_) of high-carbon fly ash, zeolite and its carbon composite, without and with adsorbates. The values of this parameter, as a function of solution pH, were calculated by the difference between the volumes of titrant added to the suspension and the supporting electrolyte solution (with a defined pH value) using the computer program “titr_v3” and the following equation (Equation (7)) [42]:

(7) $$\sigma_{0}\;=\frac{\Delta V_{\mathrm{cb}}\;F}{mS}$$

where *c_b_*—the base concentration, *F*—the Faraday constant, *m*—the solid mass in the suspension, *S*—the specific surface area of the solid, Δ*V*—the difference in the volume of base that must be added to adjust the pH of the suspension and supporting electrolyte to the specified value.

The titration set consisted of a Teflon vessel containing the solution connected to an RE 204 thermostat (Lauda), an automated microburet Dosimat 765 (Metrohm) and a computer, glass and calomel electrodes (Beckman Instruments) and a PHM 240 pH meter (Radiometer) controlling the pH values. The examined suspensions were prepared by adding 0.4 g of HiC FA, 0.4 g of Na-P1 or 0.02 g of Na-P1(C) to 50 cm^3^ of supporting electrolyte (0.001 M NaCl). The samples were titrated with 0.1 M NaCl solution in the pH range changing from 3 to 11. One by one, the supporting electrolyte itself, suspensions without adsorbates and suspensions with one or two adsorbates were titrated. The heavy metal ion concentration was 10 ppm.

Measurements of the **electrophoretic mobility** (*u_e_*) were performed to determine the zeta potential (ζ) of the particles, without and with all adsorbates, using the Henry equation (Equation (8)) [43]:

(8) $$u_{e}=\frac{2\varepsilon_{0}\varepsilon \zeta }{3\eta }f\left[\kappa \alpha \right]$$

where *ε*—the dielectric constant, *ε*_0_—the electric permeability of vacuum, *ζ*—the zeta potential, η—the solution viscosity, and *f*[*κα*]—the Henry function.

The suspensions were prepared by adding 0.01 g of HiC FA, Na-P1 or Na-P1(C) to 200 cm^3^ of the supporting electrolyte solution without and with one or two heavy metal ions, and then sonicated for 3 min. Next, the solution was divided into parts, and the pH of each of them was adjusted to a specific pH value (ranging from 3 to 10). The electrophoretic mobility measurements were carried out at an ion concentration of 10 ppm using Nano ZS Zetameter (Malvern Instruments).

## 3. Results and Discussion

### 3.1. Characteristics of Adsorbents

The chemical composition of high-carbon fly ash, carbon–zeolite composite and pure zeolite is shown in Table 1. 

The chemical composition of all adsorbents is dominated by silicon (29.72%, 18.05% and 49.8% for HiC FA, Na-P1(C) and Na-P1, respectively) and aluminum (14.11%, 10.07%; 18.05% for HiC FA, Na-P1(C) and Na-P1, respectively). In addition, there are negligible amounts of CaO (0.31–3.67%), Fe_2_O_3_ (0.51–9.12%) and Na_2_O (0.61–8.11%). Fly ash and composite are also characterized by a high carbon content, ranging from 29.65% to 44.49%. Table 2 presents the distribution of individual grain fractions of all adsorbents. 

One clearly dominant fraction was not observed in high-carbon fly ash, whereas the three main fractions 20–50, 50–100 and 100–250 µm, amounting to 27.0, 28.6 and 26.4%, respectively, were noted. The remaining fractions (equal to over 17%) are 2–20 µm (13.7%) and 250–500 µm (4.1%). The Na-P1 material is dominated by the 20–50 µm fraction, the volume content of which is 39.6%. The second is the 2–20 µm fraction (25.2%). The total volume of the particles from the particle size range of 2–100 µm is 78.1%. Obviously, particles with a diameter greater than 500 µm (3.6%) appear, which is most likely due to the fact that particles with smaller diameters agglomerate. This is typical for this type of material. In the case of the Na-P1(C) material, the total content of the 2–100 µm fraction is 78.3%, which is almost the same as for Na-P1 zeolite. However, in this case fractions 2–20, 20–50 and 50–100 µm are distributed almost evenly (25.3%, 24.7% and 28.4%, respectively) and thus, similarly to fly ash, it is difficult to unambiguously indicate the most dominant one. Additionally, the presence of particles with a diameter of 100–250 µm (18.3%) is observed, which is probably the result of the deposition of single zeolite particles on the carbon fragments, naturally increasing their size.

**Figure 1:**
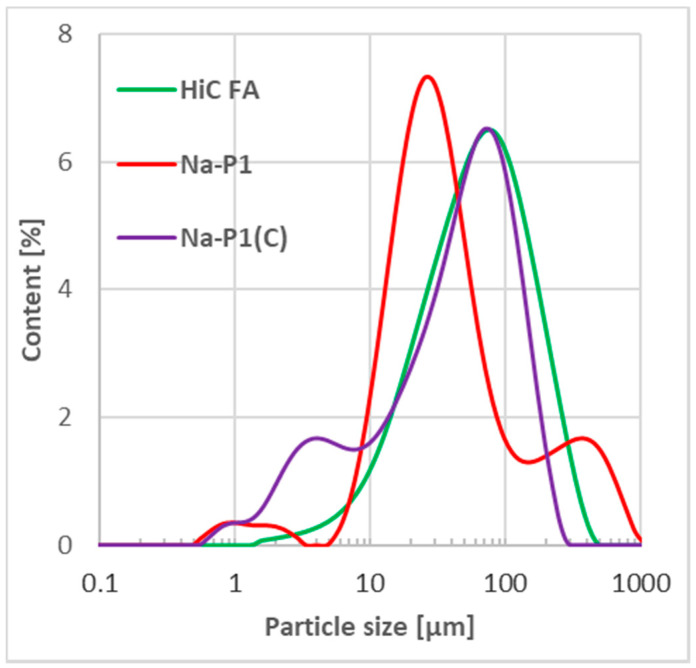
Particle size distribution of HiC FA, Na-P1 and Na-P1(C).

Figure 1 presents the curves of particle size distribution obtained for the examined adsorbents.

The particle size distribution curve obtained for the HiC FA fly ash was monomodal with a very wide peak with a maximum diameter of about 80 µm and height equal to 6.8%. The particle size distribution curves for Na-P1 and Na-P1(C) were bimodal, with easily noticeable differences in peak maxima. In the case of Na-P1, a clear maximum was observed for particles with a diameter of approx. 30 µm and a height of about 7.5%. The second maximum was noted for particles with a diameter of 400 µm (1.8%). This is consistent with the observations made for individual factions. In contrast, for the Na-P1(C) material, the maximum of the main peak was observed for particles with a diameter of about 70 µm. This peak was about 6.8%. Importantly, the peak maximum coincides perfectly with the one observed for the HiC FA fly ash (the raw material from which Na-P1(C) was obtained). The maximum of the smaller peak was near particles with a diameter of 2.5 μm. It was 1.8%, similarly to Na-P1. It can also be observed that the main peak is broad and not symmetrical, so that the smaller peak is not perfectly separated. Again, this perfectly reflects the fraction-specific observation indicating the absence of a dominant fraction (very broad main peak).

**Figure 2:**
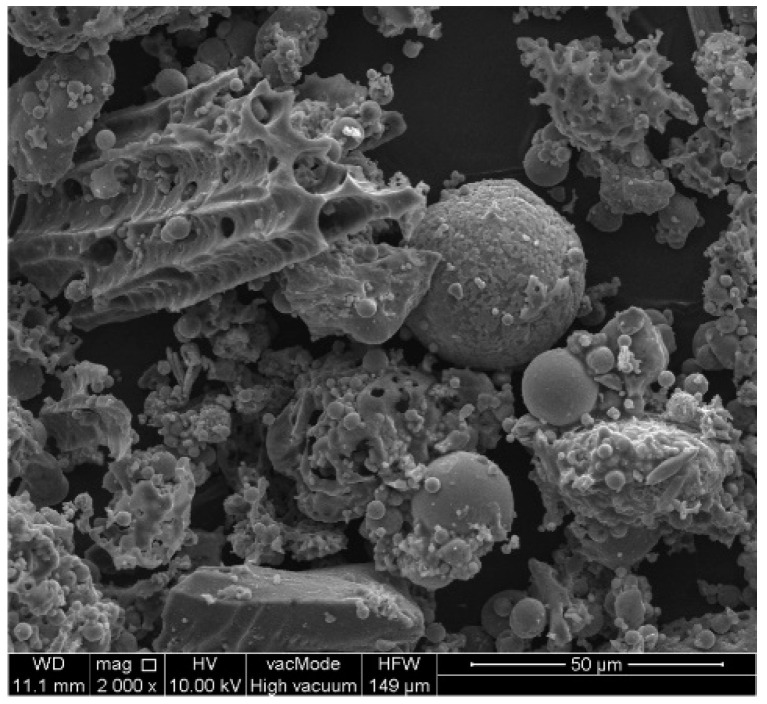
Morphological analyses of HiC FA.

**Figure 3:**
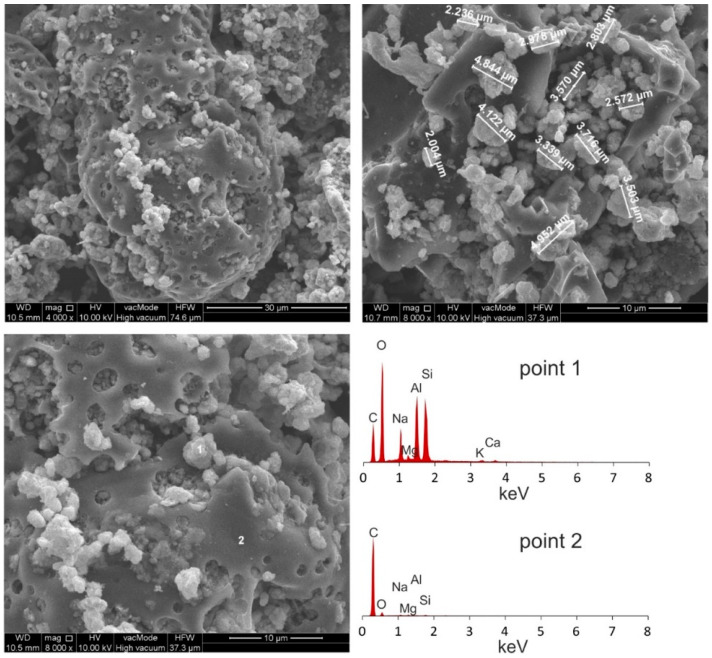
Morphological analyses of Na-P1(C).

**Figure 4:**
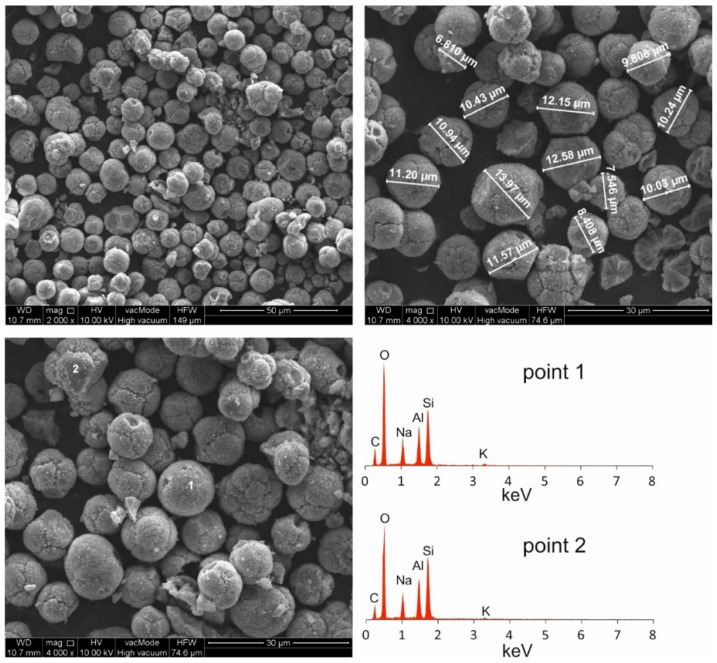
Morphological analyses of Na-P1.

Morphological analyses of high-carbon fly ash are presented in Figure 2. Figure 3 shows the morphology of the Na-P1(C), whereas Figure 4, that of Na-P1.

**Figure 5:**
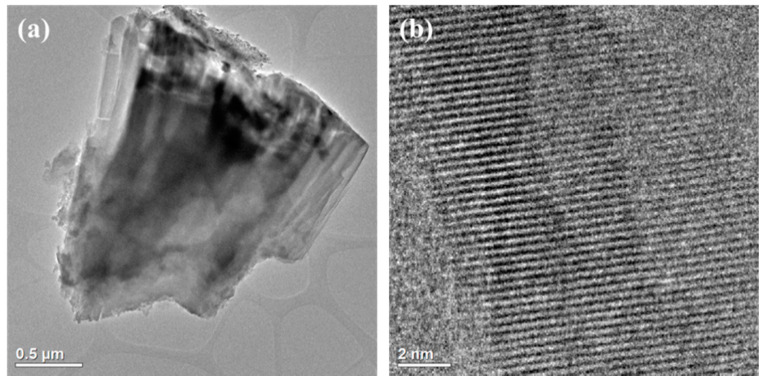
HR/TEM images of Na-P1: whole crystal (**a**), cross-section with channels (**b**).

SEM micrographs of HiC FA show spherical structures of aluminosilicate glaze, irregular forms of quartz and mullite, unburned carbon fragments and carbon sinters with embedded aluminosilicate glaze spheres. In the SEM micrographs of Na-P1(C), zeolite crystals are very well formed, creating lamellar aggregates with diameters ranging from 2 to 5 µm. These crystals are formed in the carbon ash zone as well as on fragments of aluminosilicate glaze. They have the characteristics of single clusters or mutually overgrown aggregates. Figure 3 shows the chemical analysis performed using EDS on the zeolite crystal (point 1) and the carbon fragment (point 2). In the first case, the dominant elements were aluminum, silicon, sodium, magnesium and oxygen, which are part of the zeolite, whereas in the second case, chemical analysis showed the presence of unburned carbon on which zeolite crystals were formed. In addition, some part of the carbon in the sample also came from its preparation (carbon sputtering). As was mentioned above, Figure 4 shows the morphology of pure Na-P1 zeolite (with no ash residue). The zeolite crystals are very well formed, ranging in size from 6 µm to 12 µm. This picture also shows the EDS analysis performed on zeolite crystals (points 1 and 2). In both cases, the dominant elements were silicon, aluminum, sodium and oxygen, and small amounts of calcium, potassium and magnesium. The presence of carbon was associated with the sample preparation. To obtain more information about the Na-P1 structure, HR/STEM images were taken (Figure 5a,b).

Figure 5a shows a fragment of a larger zeolite aggregate about 5 µm in size. On the other hand, Figure 5b shows a cross section of the zeolite crystal. In the microarea, one can observe pores as brighter places and their walls as darker ones. The structure of the analyzed material is characterized by well-ordered, cylindrical and regular micropores.

**Figure 6:**
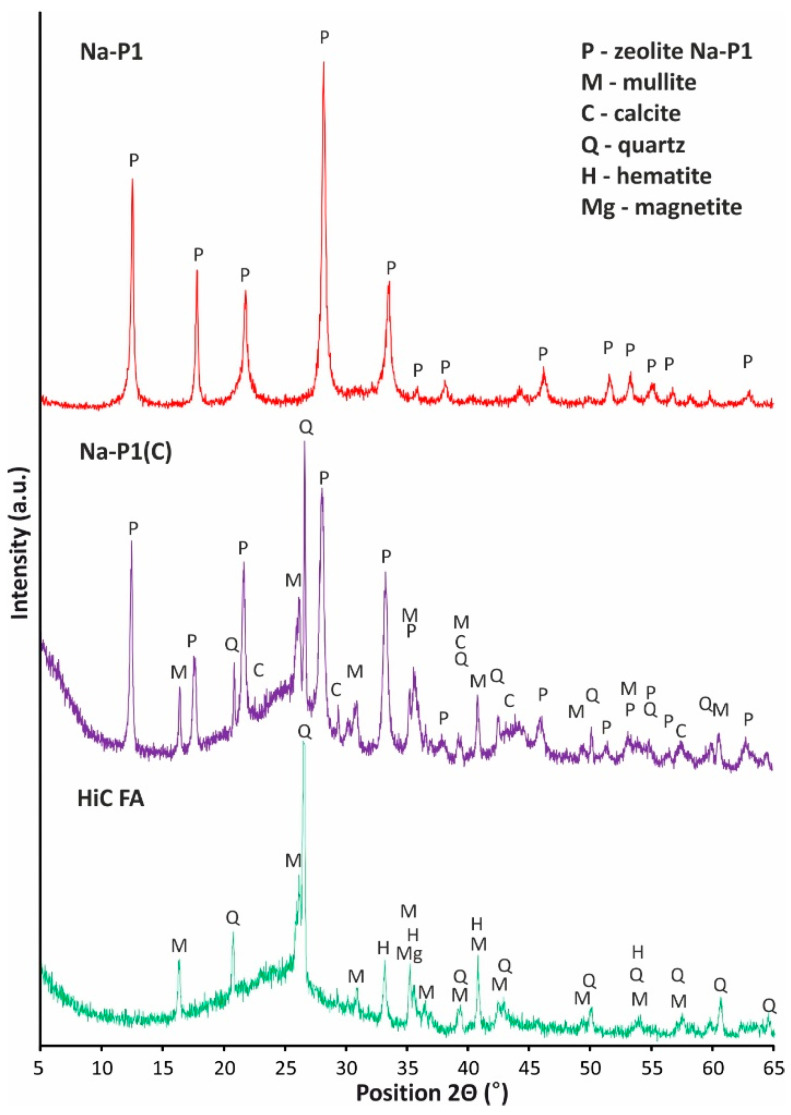
Phase analysis of high-carbon fly ash, zeolite–carbon composite Na-P1(C) and zeolite Na-P1 samples.

Diffractograms of the phase composition of high-carbon fly ash, Na-P1(C) composite and pure Na-P1 zeolite are shown in Figure 6. 

The fly ash mineral composition is dominated by amorphous aluminosilicate glaze, the presence of which is shown in the graph in the form of a raised background in the angle range 2Θ from 15 degrees to 35 degrees. The main crystalline component is mullite, recognized by the strongest reflections of interplanar distances d_hkl_ = 5.38; 3.41; 2.89; 2.66 Å. A secondary mineral component in the tested ash is quartz, recognized by the strongest reflections d_hkl_ = 4.26; 3.34; 2.45; 2.28; 1.81 Å. Moreover, the phase composition of fly ash is complemented by iron oxides in the form of hematite and magnetite, which were recognized by the strongest reflections d_hkl_ = 3.68; 2.29; 2.21; 1.6 Å. The diffractograms of the Na-P1(C) and the Na-P1 zeolite indicate that only the pure Na-P1 zeolite sample obtained from the filtrate has a monomineral character. The presence of the zeolite phase was characterized by the following reflections: d_hkl_ = 7.21; 5.05; 4.11; 3.20; 2.91; 2.69; 2.53; 2.38; 1.97 Å. The composite sample also contains phases related to unreacted ash residue in the form of aluminosilicate glaze, mullite (d_hkl_ = 5.38; 3.40; 2.79; 2.59 Å), quartz (d_hkl_ = 4.25; 3.34; 2, 45; 2.28 Å) and calcite (d_hkl_ = 3.03; 2.49; 2.09; 1.88 Å).

Textural parameters (S_BET_—specific surface area, S_micro_—micropore area, V_t_—total pore volume, V_micro_—micropore volume, and D—average pore diameter) of high-carbon fly ash, zeolite and zeolite–carbon composite are presented in Table 3.

**Figure 7:**
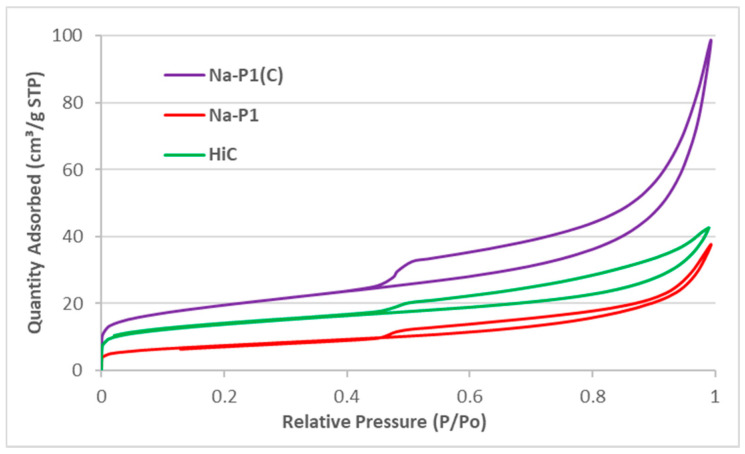
N_2_ adsorption/desorption isotherms of the studied materials.

These results indicated that all adsorbents had a poorly developed surface. They contained mesopores of average diameter in the range 5.66–6.93 nm. Figure 7 shows the nitrogen adsorption–desorption isotherms at liquid nitrogen temperature of the studied materials. It can be observed that the Na-P1(C) material is characterized by the highest nitrogen adsorption, which is confirmed by the textural parameters of these materials presented in Table 3.

### 3.2. Adsorption Capacity of HiC FA, Zeolite–Carbon Composite and Pure Zeolite Relative to Zn(II)/Pb(II) Ions in the Single Adsorbate Systems

**Figure 8:**
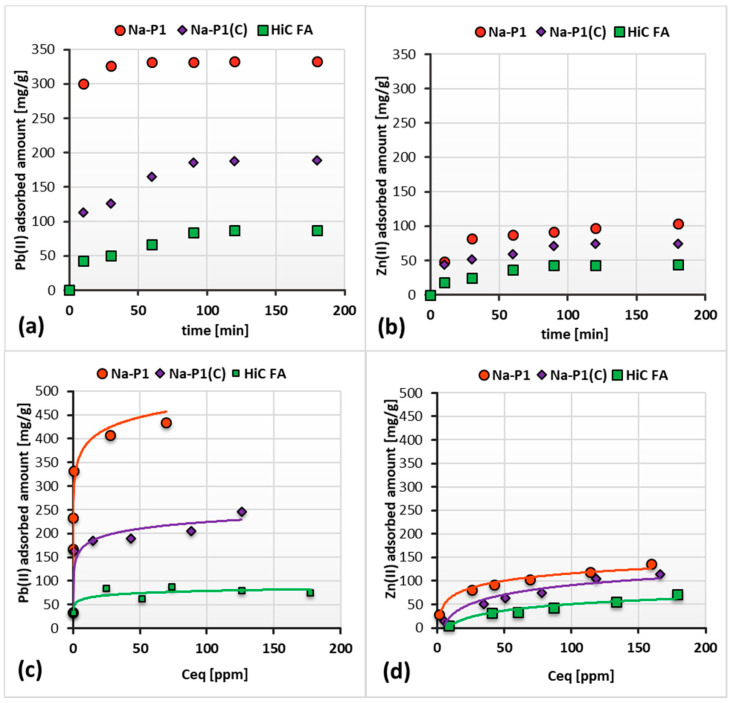
Adsorption kinetics isotherms of Pb(II) (**a**,**c**) and Zn(II) ions (**b**,**d**) on the HiC FA, Na-P1 and Na-P1(C) surfaces in the single systems at pH 5.

Figure 8a–d show the adsorption kinetics and isotherms of Pb(II) and Zn(II) ions on the HiC FA, zeolite and zeolite–carbon composite surfaces, whereas in Table 4 the kinetics and isotherm parameters are presented.

The performed measurements allowed us to determine the time after which the amount of adsorbate on the solid surface did not change. As can be seen in Figure 8a,b, this time for the fly ash, zeolite and composite differs. Among the systems containing Pb(II) ions, the equilibrium was established the fastest in the case of Na-P1, i.e., after 30 min. For Na-P1(C) and HiC FA, this time was equal to 60 min. On the other hand, among the system containing Zn(II) ions, the equilibrium was reached after 120 min in all the studied systems. Thus, it can be concluded that in systems containing Pb(II) ions, the time required to reach equilibrium is shorter than in the case of systems containing Zn(II) ions.

The observed adsorption capacities varied between selected solids, as can be seen in Figure 8c,d. Pb(II) cations are apparently better absorbed on fly ash, zeolite and its carbon composite than Zn(II) cations. The adsorbed amount of Pb(II) on the Na-P1 exceeds twice the adsorbed amount of this element on the Na-P1(C) surface, and 2.5 times the adsorbed amount on the HiC FA one. For an initial Pb(II) concentration of 150 ppm, the amount of adsorbed heavy metal is 407.34 mg/g for Na-P1, 205.00 mg/g for Na-P1(C) and 78.67 mg/g for HiC FA. Zn(II) is absorbed less efficiently. The difference in adsorption level between Zn(II) and Pb(II) cations is best seen in the case of Na-P1, where the amount of the adsorbed Pb(II) is 3.5 times larger than Zn(II). For an initial Pb(II) concentration of 150 ppm, the amount of absorbed Pb(II) ions is 407.34 mg/g, whereas the amount of absorbed Zn(II) ions is 119.33 mg/g. The significantly higher adsorption of Pb(II), compared to Zn(II), can be explained by the different affinity of these ions for zeolite, which is the result of various radii and energies of hydration of these ions. The molecular sieve effect, which is characteristic of systems containing synthetic zeolites, can explain this phenomenon. It assumes that the smaller the radius of the hydrated cation and the higher its hydration energy are, the more difficult ion penetration into the zeolite pores is. As a result, the participation of the cation in ion exchange is prevented. In the systems studied, Pb(II) has a much larger radius of the hydrated ion, which simplifies its diffusion into the pores of the zeolite and effective ion exchange with, e.g., Mg and Ca cations. As a result, a considerable increase in Pb(II) adsorption is observed [44,45].

The experimental kinetics parameters were better fitted to the pseudo-second-order model. This model assumes that the rate-controlling step is a chemical reaction—exchange or sharing of electrons between heavy metal ions and adsorbents, resulting in the formation of chemical bonds (covalent or ionic) between them. So, the Zn(II) and Pb(II) adsorption involved chemisorption. The good fit is indicated by the value of the squared correlation coefficient R^2^, shown in Table 4 [46]. Analysis of theoretical and experimental data led to the conclusion that the adsorption of both heavy metal ions is slightly better fitted to the Langmuir model than to the Freundlich one. Thus, in the systems, a monolayer of uniform energy is formed [47,48].

### 3.3. Adsorption Capacity of HiC FA, Zeolite and Its Carbon Composite Relative to Zn(II) and Pb(II) Ions in the Mixed Adsorbate Solutions

**Figure 9:**
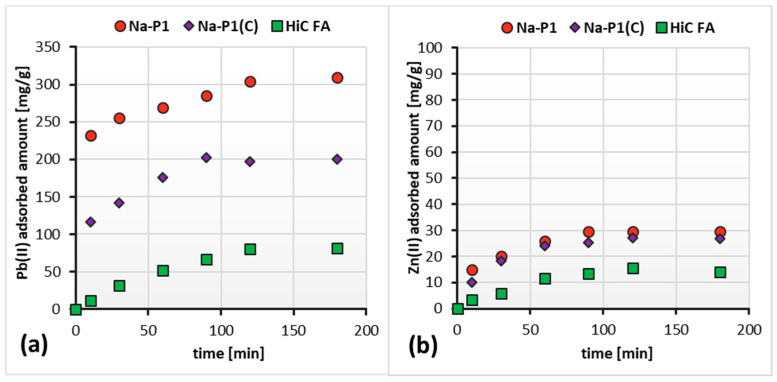
Adsorbed amounts of Pb(II) (**a**) and Zn(II) (**b**) on the HiC FA, Na-P1 and Na-P1(C) surfaces in the mixed systems as a function of time, at pH 5.

**Figure 10:**
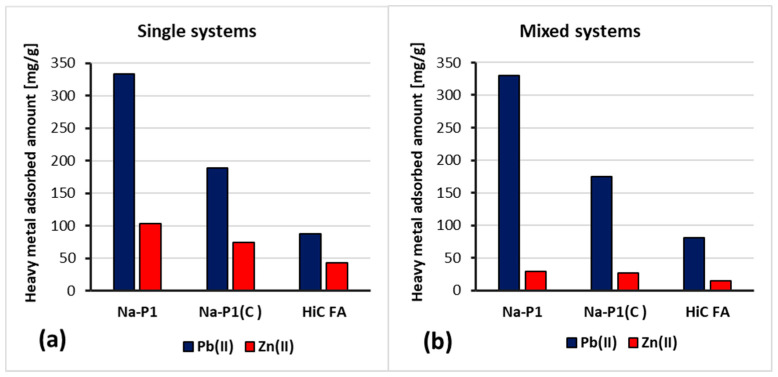
Amounts of Pb(II) and Zn(II) ions adsorbed on HCFA, Na-P1 and Na-P1(C) in the single (**a**) and mixed (**b**) systems at pH 5.

Figure 9a,b show adsorption kinetics of Zn(II) and Pb(II) ions on the HiC FA, Na-P1, Na-P1(C) surfaces in systems containing both ions simultaneously. In turn, Figure 10a,b present a comparison of the absorbed amount of Pb(II) or/and Zn(II) on the high-carbon fly ash, zeolite and zeolite–carbon composite surfaces in the single and mixed systems. 

The analysis of the obtained results showed that the simultaneous presence of Pb(II) and Zn(II) ions did not significantly affect the time of establishing the adsorption equilibrium in the studied systems. Furthermore, the amount of adsorbed Pb(II) ions on the HiC FA, Na-P1, Na-P1(C) surfaces did not change significantly since Zn(II) ions were added to the system. On the other hand, in the case of Zn(II) adsorption, the addition of Pb(II) ions to the system resulted in approximately a threefold decrease in the adsorbed amount of zinc. The reduced amount of zinc adsorbed in the mixed systems compared to the single ones may be caused by competition for adsorbents’ active sites and the aforementioned effect, resulting in a higher affinity of Pb(II) ions for three-dimensional zeolite structures.

### 3.4. Changes in Surface Charge Density of HiC FA, Na-P1 and Na-P1(C) Particles as a Result of Zn(II) and/or Pb(II) Adsorption

**Figure 11:**
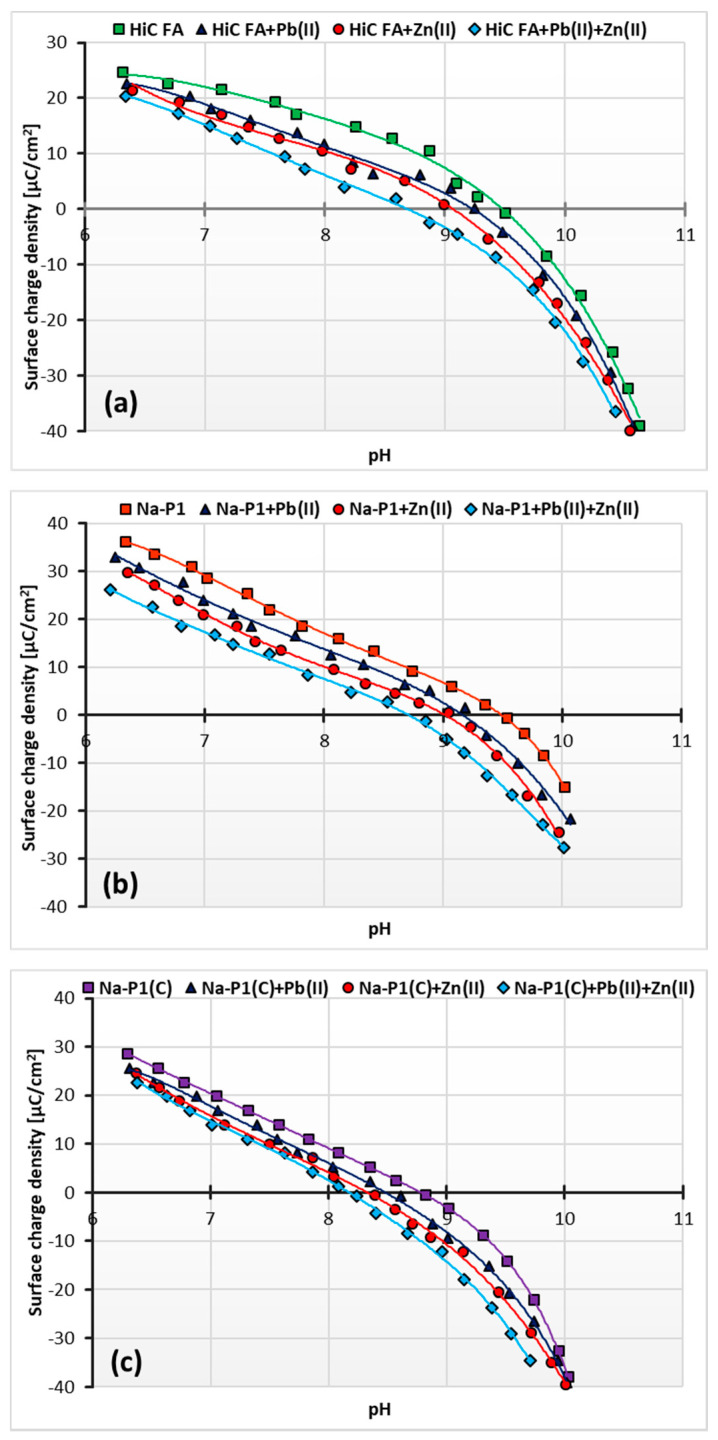
Surface charge density of HiC FA (**a**), Na-P1 (**b**) and Na-P1(C) (**c**) without and with one or two heavy metal ions.

Figure 11a–c show changes in surface charge density of the HiC FA, Na-P1 and Na-P1(C) solids as a function of solution pH, depending on the added adsorbate. In turn, Table 5 shows the changes in the pH_pzc_ (pzc—point of zero charge) values of the adsorbent particle in the examined systems of adsorbates.

As it results from the presented potentiometric titration curves, at the pH value of 5, i.e., under the conditions in which the adsorption study was conducted, the surfaces of the all adsorbents assumed a positive charge. The point of zero charge indicated the pH value at which the charge of the solid surface equals zero. At a pH higher than the pH_pzc_ of the solid, its surface takes a negative charge, and correspondingly, at a pH lower than the pH_pzc_, the surface of the solid becomes a positive charge [49]. Despite the unfavorable electrostatic repulsion at pH 5, adsorption of cations occurs on positively charged solids due to the unique structure of zeolites. They can boast a three-dimensional system of channels and chambers of strictly defined molecular dimensions, which gives zeolites the properties of molecular sieves and enables the occurrence of ion exchange. Moreover, as proved by kinetics studies, chemical bonds between simple metal ions and the solid surface are formed [50,51,52,53].

The point of zero charge is 9.5 for HiC FA, 9.6 for Na-P1, and 8.8 for NaP1(C). For all adsorbents, this parameter decreases after adding Pb(II) ions, respectively, to 9.2 for HiC FA, 9.1 for Na-P1 and 8.5 for Na-P1(C) and after the addition of Zn^2+^ ions to 9 for HiC FA and Na-P1, and to 8.4 for Na-P1(C). Moreover, in a system containing both heavy metal ions, pH_pzc_ decreases most noticeably, for HiC FA and Na-P1 to 8.6, and for Na-P1(C) to 8.3. This means that the adsorption of heavy metal ions affects the surface charge of the adsorbents, causing the pH_pzc_ to shift towards lower values, which is a commonly observed phenomenon. Adsorption of small ions (cations in this case) creates oppositely charged sites on the surface of the adsorbent. It manifests itself in a decrease of the solid surface charge density and, consequently, in a shift of pH_pzc_ position towards smaller pH values. Such a phenomenon may be described by the following equations (Equations (9)–(11)) [54,55]:

−SOH + Me^2+^ ⇌ −SO^−^ Me^2+^ + H^+^ (9)

2(−SOH) + Me^2+^ ⇌ (−SO^−^)_2_Me^2+^ + 2H^+^ (10)

−SOH + Me^2+^ + H_2_O ⇌ −SO^−^ MeOH^+^ + 2H^+^ (11)

where Me is a Pb or Zn atom.

### 3.5. Changes in Zeta Potential of HiC FA, Na-P1 and Na-P1(C) Particles as a Result of Zn(II) and/or Pb(II) Adsorption

**Figure 12:**
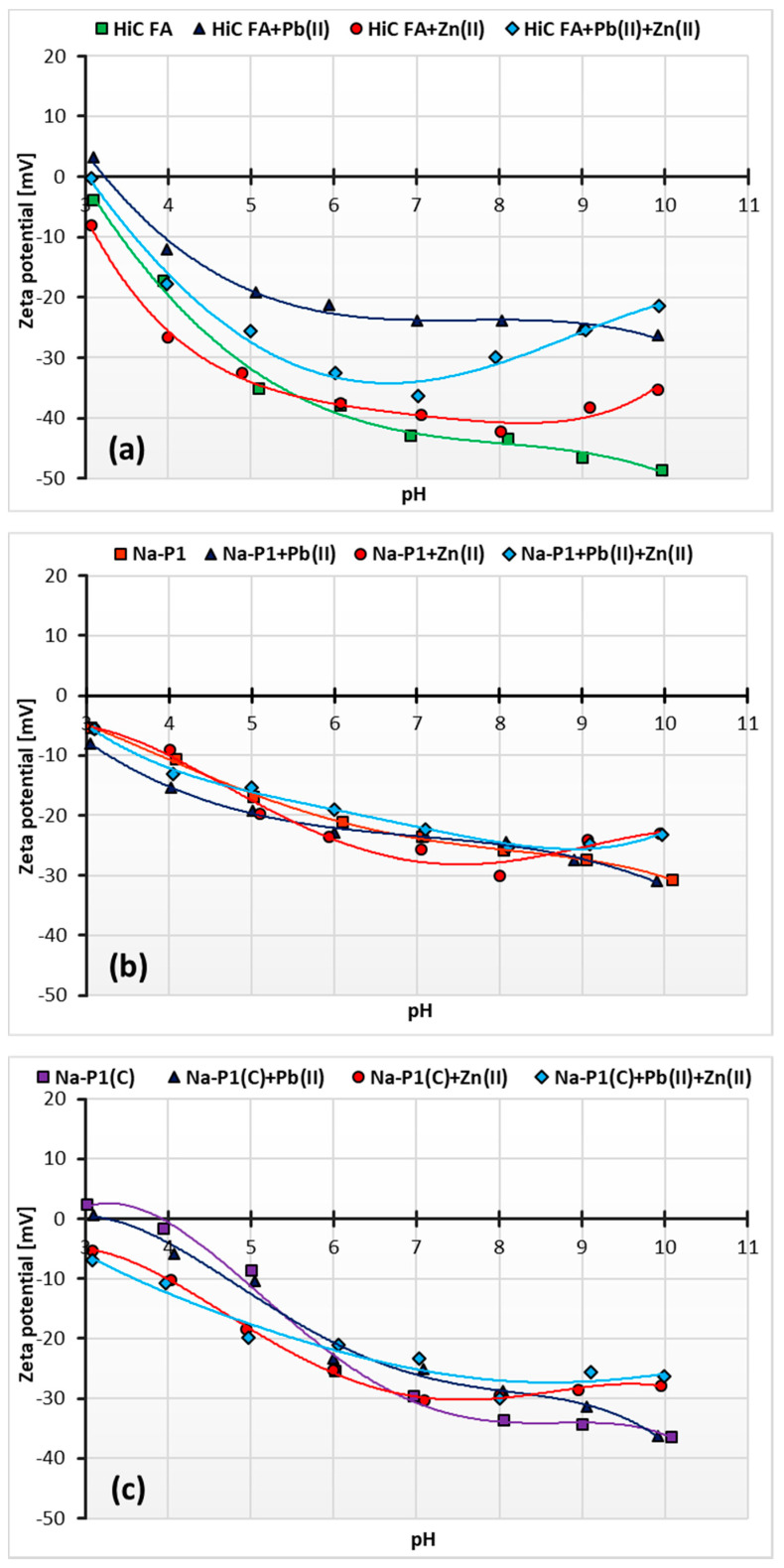
Zeta potential of the HiC FA 9 (**a**), Na-P1 (**b**) and Na-P1(C) (**c**) without and with one or two heavy metal ions.

Figure 12a–c presents the changes in zeta potential of adsorbents ions as a function of solution pH, depending on the added ions, whereas Table 6 shows the changes in pH_iep_ (iep—isoelectric point) values of the adsorbent particles, depending on the composition of the studied system.

The pH at which the charge of a slipping plane area is equal to zero is called the isoelectric point (pH_iep_). This plane is the interface separating the stiff part of the liquid “attached” to the surface of the solid (specifically and electrostatically bound water, ions and hydrated ions) from the diffuse part of the solution. Whether the pH_iep_ takes on positive or negative values is determined by the predominance of ions carrying a positive or negative charge in this plane. At pH values higher than the solid pH_iep_, the zeta potential becomes positive values, and at pH values lower than pH_iep_, negative ones [56,57]. 

The pH_iep_ values of the adsorbent particles in the studied systems are not in the range of the tested pH values. This indicates that pH_iep_ is below the value of 3. This applies, for example, to systems with Na-P1, both without adsorbates and with one or two adsorbates, as well as to Na-P1(C) (in systems with Zn(II) ions and Pb(II) and Zn(II) simultaneously) and HiC FA (without adsorbates and with Zn(II) ions). The isoelectric point of Na-P1(C) is located at pH about 4 and decreases to the value 3.2 with the addition of Pb(II) ions. For suspensions containing HiC FA, the most significant decrease in zeta potential is observed. It is manifested by a change in pH_iep_ value from 3.2 for the system with Pb(II) ions to 3 for the system with Zn(II) and Pb(II) ions. 

The specific increase in the zeta potential at pH values in the range 8–10 can be described by the effect of charge reversal, which occurs especially in the systems with Zn(II) ions. The charges of counterions in the inner part of the electrical double layer exceed the charge of ions on the solid surface, resulting in the same charge on the outer part and on the surface. The effect caused by Zn(II) ion adsorption is also called overcharging or overloading of the electrical double layer [57].

### 3.6. Desorption Degree of Zn(II) and Pb(II) from HiC FA, Na-P1 and Na-P1(C) Surfaces

**Figure 13:**
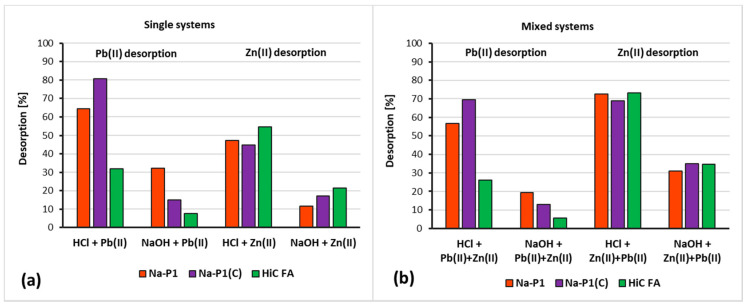
Desorption (%) of Zn(II) and Pb(II) ions from the HiC FA, Na-P1 and Na-P1(C) surfaces in single (**a**) and mixed (**b**) systems using 0.1 M HCl or 0.1 M NaOH.

Figure 13a,b presents desorption degrees for the studied adsorbents. 

The analysis of the data presented in Figure 13 leads to the conclusion that hydrochloric acid is a better desorbing agent than sodium base. This trend is present in all suspensions studied, both for single and mixed systems, regardless of the adsorbate or adsorbent used. The most efficient desorption from the surface of zeolite and zeolite–carbon composite in single systems took place with Pb(II) ions, whereas the greatest desorption from HiC FA surface occurred in systems containing Zn(II) ions. In mixed systems, these trends were similar.

## 4. Conclusions

Two types of sorbents were obtained from the waste that is fly ash—a zeolite–carbon composite and pure zeolite. Both zeolite and its carbon composite showed significantly better adsorption capacities than their precursor, fly ash. This is a result of their better textural and morphological parameters obtained due to the conversion of fly ash (zeolite–carbon composite) and waste solution (pure zeolite). Pb(II) ions were adsorbed more efficiently than Zn(II) ones and their adsorption occurs fast. The largest adsorption takes place on the Na-P1 surface (407.34 mg/g for Pb(II)), whereas the smallest on the HiC FA one (78.67 mg/g for Pb(II)). Bounding of all adsorbates occurs through chemisorption and ion adsorption following the Langmuir model. In mixed systems, Pb(II) ions adsorb more efficiently due to the molecular sieving effect. In the mixed systems of adsorbates, the presence of Zn(II) ions minimally influences the adsorbed amount of Pb(II) ions. On the contrary, the addition of Pb(II) ions to the system results in a threefold decrease in the adsorbed amount of Zn(II) ions. The addition of divalent heavy metal cations decreases the pH_pzc_. More efficient desorption of heavy metal ions (reaching 80%) occurs with the use of hydrochloric acid (in comparison with sodium base). Both the composite and pure zeolite, due to the method of their synthesis, may be an alternative to commercial sorbents in terms of heavy metal ion removal from aqueous solutions. Such use of fly ash is in line with the current ecological trends.

## Figures and Tables

**Table 1:** Chemical composition of adsorbents: high-carbon fly ash, zeolite–carbon composite and zeolite.

	Compounds											
	Na_2_O	MgO	Al_2_O_3_	SiO_2_	P_2_O_5_	SO_3_	K_2_O	CaO	TiO_2_	Fe_2_O_3_	LOI	C
HiC FA	0.61	1.52	14.11	29.72	0.32	1.16	2.28	3.67	0.95	8.82	36.82	29.65
Na-P1(C)	3.77	1.19	10.07	18.05	0.04	0.53	0.42	2.17	0.91	9.12	53.73	44.49
Na-P1	8.11	nd *	18.05	49.38	nd *	nd *	5.57	0.31	0.06	0.51	17.37	nd *

nd *—not detected.

**Table 2:** Percentage distribution of individual grain fractions (µm) of HiC FA, Na-P1 and Na-P1(C).

Fraction [µm]	HiC FA	Na-P1	Na-P1(C)
	[%]		
0.01–2	0.18	2.57	3.16
2–20	13.69	25.23	25.28
20–50	27.02	39.56	24.65
50–100	28.64	13.29	28.41
100–250	26.41	8.34	18.34
250–500	4.05	7.34	0.17
500–1000	0	3.64	0
1000–2000	0	0.04	0

**Table 3:** Textural parameters of HiC FA, Na-P1 and Na-P1(C) adsorbents.

Adsorbent	S_BET_ [m^2^/g]	S_micro_ [m^2^/g]	V_t_ [cm^3^/g]	V_micro_ [cm^3^/g]	D (4 V/A) [nm]
HiC FA	46.19	10.72	0.064	0.0060	5.66
Na-P1	26.71	4.30	0.05	0.0017	6.93
Na-P1(C)	69.85	16.90	0.12	0.0070	6.75

**Table 4:** Kinetics and isotherm parameters of Pb(II) and Zn(II) adsorption on the HiC FA, Na-P1 and Na-P1(C) surfaces at pH 5.

System		Pseudo-Second-Order Model		
		q_e_ [mg/g]	k_2_ [g/(mg·min)]	R^2^
Pb(II)	Na-P1	322.581	0.0004	0.997
	Na-P1(C)	212.766	0.0004	0.997
	HCFA	129.870	0.0001	0.959
Zn(II)	Na-P1	32.362	0.0023	0.996
	Na-P1(C)	30.030	0.0019	0.998
	HCFA	19.083	0.0011	0.940
**System**		**Langmuir model**		
		**q_m_** **[mg/g]**	**K_L_** **[dm^3^/mg]**	**R^2^**
Pb(II)	Na-P1	432.572	3.755	0.999
	Na-P1(C)	236.661	0.248	0.978
	HCFA	76.548	2.911	0.988
Zn(II)	Na-P1	144.062	0.053	0.980
	Na-P1(C)	156.988	0.014	0.962
	HCFA	142.192	0.007	0.860

**Table 5:** Comparison of the pH_pzc_ values of HiC FA, Na-P1, Na-P1(C) particles in the absence or presence of heavy metal ion/ions.

Solid	Na-P1	Na-P1(C)	HiC FA
Without adsorbates	9.6	8.8	9.5
With Pb(II)	9.1	8.5	9.2
With Zn(II)	9.0	8.4	9.0
With Pb(II) + Zn(II)	8.6	8.3	8.6

**Table 6:** Comparison of the pH_iep_ values of the HiC FA, Na-P1, Na-P1(C) particles in the absence or presence of heavy metal ion/ions.

Solid	Na-P1	Na-P1(C)	HiC FA
Without adsorbates	-	4.0	-
With Pb(II)	-	3.2	3.2
With Zn(II)	-	-	-
With Pb(II) + Zn(II)	-	-	3.0

## Data Availability

The data presented in this study are available on request from the corresponding author.
